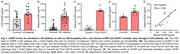# Extracellular CIRP induces neurotoxic A1 astrocytes via TREM‐1 in Alzheimer’s disease

**DOI:** 10.1002/alz.094835

**Published:** 2025-01-09

**Authors:** Archna Sharma, Dilara Aylar, Yongchan Lee, Max Brenner, Philippe Marambaud, Ping Wang

**Affiliations:** ^1^ Feinstein Institutes for Medical Research, Manhasset, NY USA

## Abstract

**Background:**

While A1 astrocytes are reported in Alzheimer’s disease (AD), the underlying molecular mechanisms are complex and remain elusive. Proinflammatory extracellular cold‐inducible RNA‐binding protein (eCIRP) is released by microglia in response to AD‐associated neuronal amyloid‐β. eCIRP activates the triggering receptor expressed on myeloid cells‐1 (TREM‐1). Thus, we hypothesized that increased levels of eCIRP in AD due to inflammatory stress induce A1 via astrocytic TREM‐1.

**Method:**

ELISA was performed in the AD patients’ cerebrospinal fluid (CSF; in‐house Alzheimer’s Disease Center repository) or plasma (Precision Biospecimen Solutions, Bethesda, MD) or hTau.P301S mice (Dr. Michel Goedert, Cambridge, UK) plasma. C8‐D1a cells (ATCC) or magnetically purified primary astrocytes, from C57BL/6 mice ± small anti‐CIRP peptide M3 or from TREM1‐KO mice, were stimulated with eCIRP. C57BL/6 mice were injected intracerebroventricular (*icv*) with eCIRP. Expression of mRNA was quantified by qPCR, protein by immunoblotting and released levels by ELISA or R&D proteome profiler. Student’s *t‐test* was used for two‐group analysis, one‐way analysis of variance (ANOVA)‐SNK test for multigroup analysis, and Spearman’s‐Rho analysis for correlations, with significance if *p* < 0.05.

**Result:**

Levels of eCIRP were elevated in the CSF and plasma of AD patients, in hTau.P301S mice, and plasma eCIRP strongly correlated with astrocyte activation marker glial fibrillary acidic protein (GFAP) in AD patients (**Fig. 1**). eCIRP strongly induced A1 astrocyte‐specific genes and astrocyte release of proinflammatory and neurotoxic factors in C8‐D1a cells, primary astrocytes and *icv* eCIRP‐injected mice brains. In particular, eCIRP increased Complement 3, an A1 astrocytic marker involved in neuroinflammation‐associated neurodegeneration. Primary astrocytes from TREM‐1 knockout mice were resistant to eCIRP induction of A1 astrocytes. Moreover, astrocytes exposed to eCIRP had higher TREM‐1 total and surface protein levels, increased Syk phosphorylation, and upregulated NFκB mRNA. Importantly, M3 effectively inhibited eCIRP’s induction of A1 astrocytes and their release of proinflammatory and neurotoxic mediators.

**Conclusion:**

Collectively, our data provides a strong correlation of eCIRP with astrocyte reactivity in AD patients, identifies a novel molecular mechanism by which eCIRP induces A1 astrocytes via TREM‐1 and M3 peptide attenuation of eCIRP‐induced A1 astrocytes. These findings point to a novel therapeutic opportunity targeting neurotoxic A1 astrocytes in AD.